# Three-Dimensional Assessment of Mandibular Condylar Volume and Position Subsequent to Twin Block Functional Therapy of Skeletal Class II Malocclusion Accompanied by Low-Level Laser Therapy

**DOI:** 10.3390/dj8040115

**Published:** 2020-10-09

**Authors:** Mahmoud Abdel Hameed Mohamed, Khaled Farouk Abdallah, Farouk Ahmed Hussein

**Affiliations:** Department of Orthodontics, Faculty of Dental Medicine (Boys), Al-Azhar University, Cairo 11651, Egypt; Mahmoudhelaly.209@azhar.edu.eg (M.A.H.M.); Khaledfarouk@azhar.edu.eg (K.F.A.)

**Keywords:** low-level laser therapy, twin block, skeletal class II malocclusion, condylar volume and position, cone beam computed tomography

## Abstract

This study aimed to evaluate and compare the effect of low-level laser therapy (LLLT) on mandibular condylar volume and position following treatment of a Class II malocclusion with a twin block (TB) appliance employing cone beam computed tomography (CBCT). Twenty-four growing patients, aged 9–12 years, were randomly allocated into control and laser groups. All patients were treated with a TB appliance. The patients in the laser group were treated weekly with a gallium–aluminum–arsenide (GaAlAs) diode laser around the temporomandibular joint (TMJ) region for three months. CBCT images were obtained before and after TB therapy and the changes in TMJ and skeletal variables were evaluated and compared among and between the groups. In the laser group, the condylar volume of the right and left sides significantly increased by 213.3 mm^3^ and 231.2 mm^3^, respectively (*p* < 0.05), whereas in the control group it significantly increased by 225.2 mm^3^, and 244.2 mm^3^, respectively (*p* < 0.05), with forward and lateral positioning of both sides. Furthermore, effective mandibular, ramus, and corpus lengths were increased, which were not significant between the groups. Low-Level Laser therapy accomplished no considerable effect on mandibular condylar volume and position following the functional orthopedic treatment of skeletal Class II malocclusions using a TB appliance.

## 1. Introduction

Class II malocclusions are seen as the most frequent skeletal problems in the orthodontic community and constitute a significant proportion of orthodontic patients ranging from 18% up to approximately 32%. It has been claimed that these malocclusions are often due to a mandibular deficiency or retrognathism. In the management of growing patients, different removable or fixed functional appliances have been recommended to stimulate or redirect the mandibular growth to improve this skeletal disparity [1,2]. However, the effect of these appliances on mandibular and/or condylar growth is debatable, and the methods by which the probable changes are triggered is still not clearly understood [2,3,4,5].

Seemingly, the efficacy of mandibular orthopedic management relies on the ultimate synergy between treatment and growth, particularly in patients who are in their pubertal growth spurt [6]. Furthermore, its possible effects regarding the temporomandibular joint (TMJ) are not obvious, with augmented interest in this area. It was proposed that these appliances could bring about a change in TMJ, which might occur due to the adaptive remodeling of condylar cartilage and glenoid fossa, thus affecting the condylar position [7,8,9,10,11,12,13,14,15,16].

In the orthodontic literature, the proposed changes in TMJ after functional therapy have been envisioned with techniques such as cephalometric and panoramic radiographs. However, limitations do exist with the image acquisition of TMJ using these methods. Technological advancements in imaging have led to the application of cone beam computed tomography (CBCT) for acquiring such images. CBCT images have a high resolution and minimal distortion, thereby allowing the creation of three-dimensional (3D) images for precise analysis with no overlapping of bilateral structures. Additionally, it has been repeatedly employed for the assessment of craniofacial morphology, dental and maxillofacial pathologies, and for mandibular condylar volume estimation [9,16,17,18].

One of the most essential factors in mandibular advancement through functional appliances is to provide condylar cellular activity in a shorter treatment time. Indeed, the duration of functional therapy relies upon several factors such as the individual’s skeletal age, gender, the severity of skeletal problems, and the appliance type. The most frequent concern for patients undergoing functional appliance therapy is the duration of treatment, which may extend up to 24 months [4,5]. Hence, there is a need for non-invasive approaches to accentuate the mandibular growth with little or no potential side effects in a short duration. The advancement in technologies capable of stimulating growth potential could allow clinicians to predictably accelerate the growth phenomena of the mandibular cartilage [19].

In experimental reports, some researchers considered innovative methods for the stimulation of condylar growth, with or without functional appliances such as ultrasound and laser applications. There is increasing documentation on the compensatory growth that occurs at the mandibular condyle in response to functional appliance therapy that is stimulated by low-intensity pulsed ultrasound [19,20]. In recent years, the application of low-level laser therapy (LLLT) has gained considerable recognition in medical and dental fields and is being utilized as a non-invasive tool to accelerate orthodontic tooth movement with no reported clinical side effects. Interestingly, LLLT has been demonstrated to promote bone healing following fracture and mandibular distraction osteogenesis [21,22]. In early experimental studies, LLLT demonstrated stimulatory effects on chondroblastic proliferation [23].

Recently, several authors examined the biostimulatory effect of LLLT on chondrocytic cultures, and revealed that it augmented cellular proliferation [24,25,26,27,28]. To the best of our knowledge of the most current and available literature, no clinical studies have evaluated the effect of LLLT on TMJ parameters during functional orthopedic treatment of Class II malocclusion patients with mandibular retrognathism.

Therefore, the present randomized clinical trial aimed to evaluate and compare the effect of accompanying LLLT on mandibular condylar volume and position following the treatment of a Class II malocclusion with a twin block (TB) appliance. It was hypothesized that the changes subsequent to twin block therapy, with and without LLLT, are not significantly different.

## 2. Materials and Methods

Ethical aspects and study design: The objectives of the study and the proposed treatment plan were explained to the patients and their guardians, and informed consent was obtained before commencing the study. The study was conducted according to the principles of the Declaration of Helsinki and the study protocol was approved by the Research Ethics Committee of Al-Azhar University, Cairo, Egypt, on 29/01/2017, with Registration No. orthod._10 Med.Research._LLLT.TMJ, Class II, _0000010. The study is registered at ClinicalTrials.gov (ID.04376190).

This study was designed as a randomized controlled clinical study with two parallel groups. A total of 24 patients (12 boys and 12 girls), aged 9–12 years, were recruited from the Out Patient Department (OPD) clinic at the Orthodontic Department, Faculty of Dental Medicine (Boys), Al- Azhar University, Cairo, Egypt. Participants were recruited after their recall from the OPD waiting lists in preparation for orthodontic treatment from January 2018 to August 2019.

Sample size estimation: According to previous studies [9,16] a sample size calculation was undertaken with statistical software (G*power version 3.1 Faul, Erdfelder, Lang and Buchner, Germany) based on the following pre-established parameters: 80% power, sample size for unpaired *t*-test, and significance level (α) = 0.05 (two-tailed).

Eligibility criteria: Patients were recruited based on the following inclusion criteria: skeletal Class II (ANB > 4°) with normal maxilla (SNA° = 82° ± 2°) and retrognathic mandible (SNB° < 78°); patients at CS2-CS3 that show initiation and peak of growth spurt to achieve maximum treatment effects of TB appliances based on the modified cervical vertebral maturation stages (CVMS) [29]; overjet > 5 mm; minimal crowding in dental arches (≤3 mm); average inclination or slightly retroclined lower incisors. The following exclusion criteria were applied: previous orthodontic treatment; severe maxillary transverse deficiency or posterior cross bites; severe facial asymmetry confirmed by clinical or radiographic examination; poor oral hygiene; any systemic diseases that may hinder the outcome of orthodontic treatment.

Randomization: All participated patients were treated with a TB appliance as the first stage for skeletal Class II malocclusion patients with mandibular retrognathism [3,5]. The patients were randomly divided into control and laser groups, involving 14 subjects in each group. The process of randomization and group allocation followed a 1:1 ratio and clinical assistants allocated patients into two groups using a computerized randomization software, http://www.graphpad.com/quickcalcs/index.com. In the laser group, patients were treated with a TB appliance combined with LLLT, whereas in the control group, patients were treated with only a TB appliance.

Orthodontic records: For each patient enrolled in the study, detailed clinical examination and routine orthodontic records were obtained initially and after treatment. In addition, CBCT images were obtained before (T1) and after completion of the orthopedic phase (T2).

Functional orthopedic appliance: All patients received a TB functional appliance according to the design developed by Clark [30] that comprised maxillary and mandibular acrylic bite blocks (Dentaurum, Orthodontic Orthocryl Clear Acryl Resin, Germany) with 70° occlusal inclined planes [3,31]. The patients were instructed to wear the TB appliance 24 h a day except during meals. All the patients were assessed once a month. During this follow-up appointment, the anteroposterior dental arch relationship was checked with and without the TB appliance. Patient cooperation was estimated according to the diary notes and signs of use. Inter-occlusal acrylic trimming was performed for all patients to allow the thorough vertical development of buccal segments in mandibular arch [9]. The overall functional treatment period was determined to be nine months [9,12,31,32].

**Figure 1:**
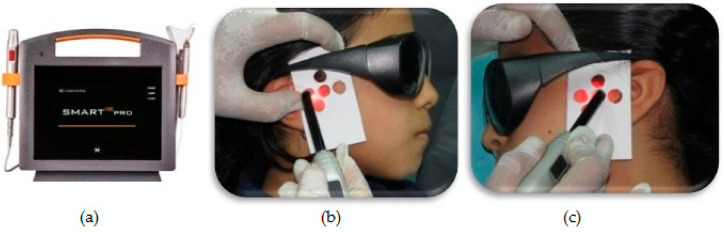
(**a**) Gallium–aluminum–arsenide semiconductor diode laser equipment used in study. Application of low-level laser therapy (LLLT) at five points within temporomandibular joint (TMJ) area on both sides for a female patient. Right side (**b**); left side (**c**).

Low level laser therapy (LLLT): In the laser group, patients received LLLT from a semiconductor gallium–aluminum–arsenide ((GaAlAs) diode laser (SMART^M^PRO, LASOTRONIX, Poland). The following parameters for LLLT were applied: 50 mw power output with 635 nm wavelength in continuous mode, 4.5 Joules/cm^2^ energy density, 11.25 J total dose per side with 45 s at a point, employing an 8-mm fiber optic tip. The laser was applied to five different areas (lateral, superior, anterior, posterior, and posterior–inferior points) located within the TMJ region on both the right and left sides and in contact with the skin, as shown in Figure 1. Laser therapy was performed every week for three months according to an established protocol [33]. All safety precautions were taken during laser application where both patient and operator used appropriate protective glasses specific to the used wavelength. All clinical treatment procedures and laser application were performed by a single investigator (M.A.H.M).

Study measurements and data collection: The primary outcome of the present study was to assess the effect of TB functional therapy, with and without LLLT, on TMJ parameters, with particular orientation to condylar volume and condylar head position. CBCT images were obtained before and following nine months of TB therapy in both groups in line with previous studies [9,15]. The CBCT scans were obtained before (T1) and after (T2) TB functional therapy with and without LLLT using a CBCT machine. A scout view was acquired, and adjustments were made to assure that all patients were correctly aligned in the scanner in accordance with the light beam prior to image acquisition. The CBCT images were obtained with an i-CAT next-generation CBCT scanner (Imaging Sciences International, Hatfield, PA) with the following exposure parameters: 20.1 cm (diameter) × 17.3 cm (length) × 10.2 cm (height) field of view, 90-kVp tube voltage, 12.5 MA, 15-s scan time, 0.3 mm voxel size, and a total dose of 2.078 ± 0.002 mGy. The data obtained were exported to Digital Imaging and Communication in Medicine (DICOM) format. Serial steps were followed by the single examiner (M.A.), who was blinded to the study groups, so as to standardize the measurements in all scans. An identification number was assigned to each CBCT image prior to analysis to mask the patient’s name and the time point during analysis. The CBCT images were re-matched to the patient’s name after data collection was completed.

**Figure 2:**
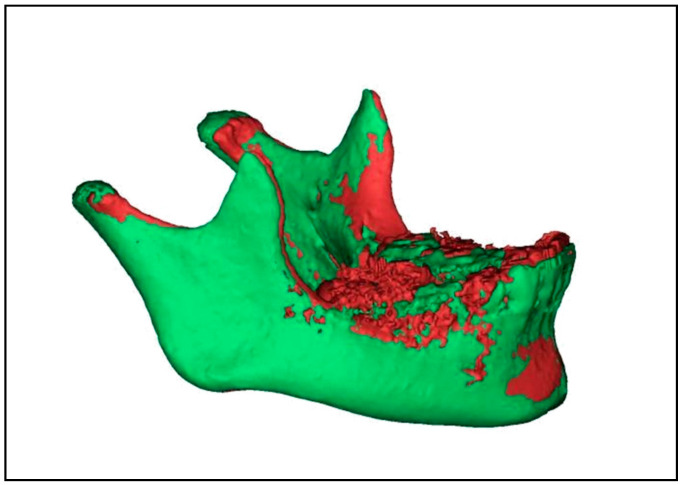
Mandibular 3D cone beam computed tomography (CBCT) image showing superimposition of before (purple) and immediately after twin block (TB) therapy (light green) images by software used in the study.

**Figure 3:**
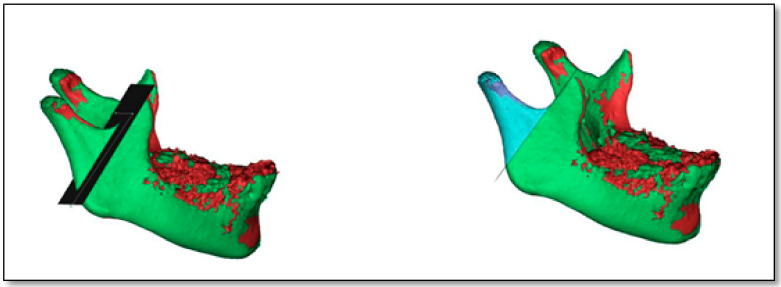
CBCT mandibular 3D images showing construction of plane passing tangent to distal slope of coronoid process to slice right and left condyles of superimposed images using Mimics software.

Assessment of condylar head volume: Both T1 and T2 mandibular 3D CBCT images were segmented for superimposition and condylar volumetric measurements via special software (Mimics software v. 16.0, Materialise NV, Leuven, Belgium). The assessment method was in accordance with the previous studies by Bayram et al. [17] and Yildirim et al. [16]. This was performed by the “superimposition” function module of the software, which was a rigid body registration in accordance with landmark fitting. Foramen mentale, superior and posterior points of antegonial notches were used on both the right and left sides (Figure 2). Next, the custom planar osteotomy function of the software was used to establish a plane of 0.1 mm passing tangentially to the distal slope of the coronoid process, thereby separating the condyles of superimposed images (Figure 3). Subsequently, after segmenting the superimposed condylar images, condylar volumetric measurements were automatically calculated in mm^3^. The same process was employed for both right and left condyles in each patient.

**Figure 4:**
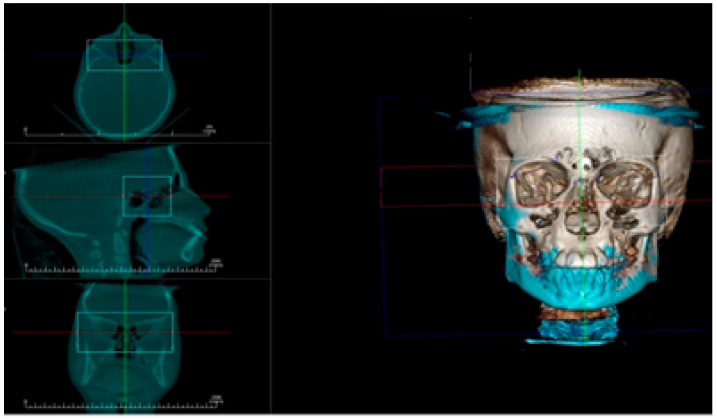
Automatic volume-based registration for superimposition of T1 and T2 CBCT images utilized in the study. Orthogonal planes: axial, coronal, and sagittal aligned as reference planes for analysis of condylar head position, Vertical Plane (VP, blue line), Frankfort Horizontal Plane (FHP, red line) and midsagittal plane (MSP, green line).

Assessment of condylar head position: The DICOM files were imported to a piece of software (Invivo Dental Anatomage; Application v 5.3.1, San Jose, CA, USA) and the orientation of each scan was rechecked and adjusted if necessary. To obtain an accurate evaluation of changes in condylar head positions between T1 and T2 images in both groups, a CBCT superimposition was made to ensure that we were referencing all measurements to the same reference planes. The three (axial, coronal and sagittal) orthogonal planes were realigned to represent the Frankfort Horizontal Plane (FHP), the Vertical Plane (VP) and midsagittal plane (MSP), respectively. From the “Superimposition” tab, the “Import New Volume” option was chosen, and DICOM files of T2 data were selected. At first, point-based registration in the midfacial region and cranial vault were employed to approximate the two scans. Next, high-precision automatic volume-based registration was utilized for the correct superimposition of T1 and T2 images (Figure 4).

The following reference landmarks and planes were used for the analysis of condylar head position:

(1) Condylion superius (CdS) point: The most right or left superior midpoint on the condylar head.
(2) Condylion anterior (CdA) point: The most right or left anterior point on the condylar head.
(3) Condylion medialis (CdM) point: The most right or left medial point on the condylar head.
(4) Frankfort Horizontal plane (FHP): Plane defined by three landmarks: right porion, left porion and left orbitale.
(5) Midsagittal plane (MSP): Plane passing through sella and nasion points and perpendicular to FHP.
(6) Vertical plane (VP): Plane passing through sella point and perpendicular to FHP and MSP.

**Figure 5:**
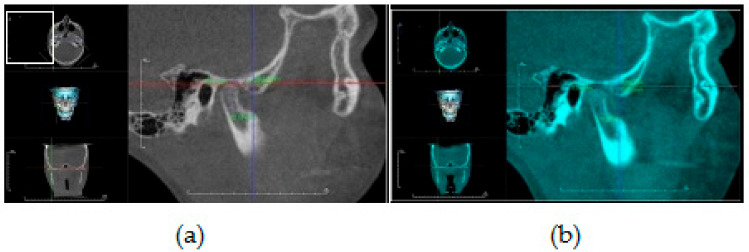
CBCT sagittal generated view showing assessment of condylar head position relative to VP (blue line) and FHP (red line): (**a**) before (T1) TB therapy; (**b**) after TB therapy (T2).

**Figure 6:**
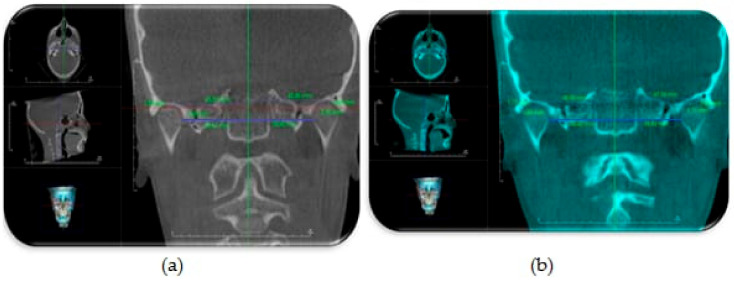
CBCT coronal generated view showing assessment of condylar head position relative to MSP (green line): (**a**) before TB therapy; (**b**) after TB therapy.

The evaluation of the condylar head position was done according to the following linear measurements (Figure 5 and Figure 6) [13]:

1. Vertical condylar position: the perpendicular distance between CdS point and FHP
2. Anteroposterior condylar position: the perpendicular distance between CdA point and VP
3. Mediolateral condylar position: the perpendicular distance between CdM point and MSP

**Figure 7:**
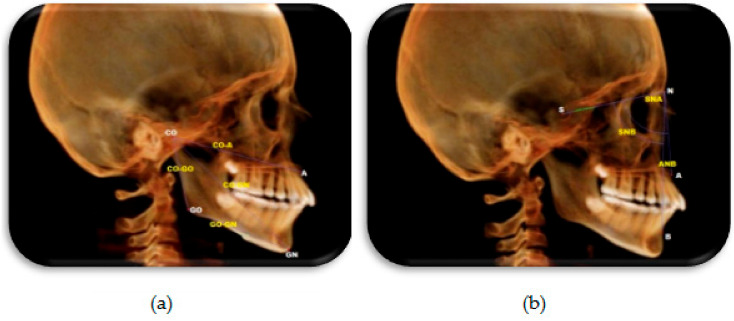
Constructed lateral cephalometric radiograph showing skeletal landmarks and measurements used in the study. (**a**) Sagittal measurements. (**b**) Angular measurements.

Assessment of skeletal changes: Lateral cephalometric radiographs were reconstructed from CBCT images to evaluate changes in the skeletal parameters in both groups, with and without LLLT. The definitions of landmarks, reference planes, and sagittal linear, angular and cephalometric measurements were defined and are shown in Table 1 and Figure 7 [2,9,14].

Statistical Analysis: Data were collected, coded and analyzed with the Statistical Package for Social Science software for windows (IBM SPSS, Version 23, Chicago, IL, USA). The distribution of quantitative data was tested by Kolmogorov–Smirnov and Shapiro–Wilk tests of normality. The results show that the data were normally distributed and, consequently, parametric tests were applied for statistical evaluation. Data were statistically described as the mean, standard deviation, 95% confidence intervals, mean standard error, and mean differences (T1-T2 changes). All data were then checked for pre-treatment equivalence between two groups with an independent sample *t*-test. A paired *t*-test was used to compare the changes within each group before (T1) and after TB therapy (T2). Additionally, an independent sample *t*-test was used to compare the difference between studied groups before and after TB therapy for both TMJ and the skeletal parameters. The independent sample test was used to compare the amount of changes (T1-T2) in the analyzed variables between the groups. The significance level was set at *p* ≤ 0.05.

## 3. Results

### 3.1. Participant Flow

**Figure 8:**
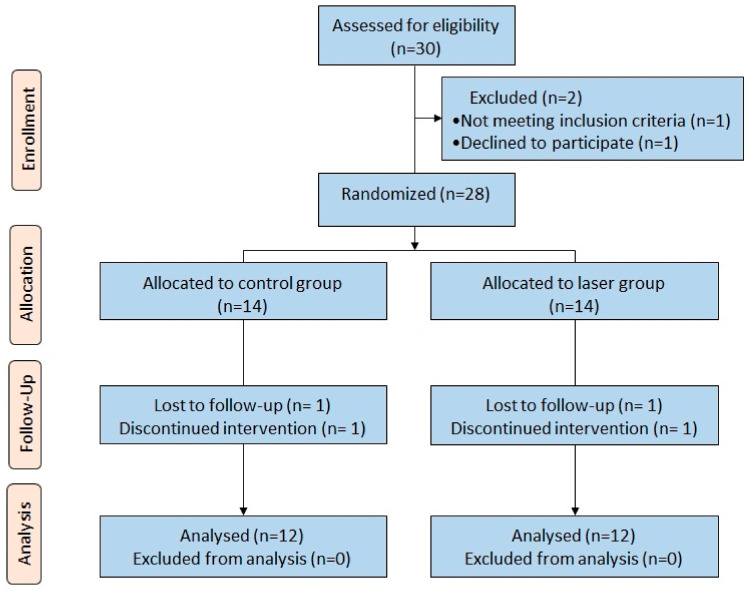
Flow chart of the study design.

The study was a randomized controlled clinical trial with two parallel groups. The study initially included 28 patients, 14 females and 14 males. However, two patients from each group (two males and two females) were lost to further intervention during the study. The study design is presented as a flow chart in Figure 8.

### 3.2. Analysis of Error of Measurements

The intra-rater reliability of the measurements was performed after four weeks and involved the analysis of eight CBCT images (repeated data of 30% of the sample) that were chosen at random from both groups. Systematic error was assessed with a paired *t*-test, and random error was assessed with the coefficient of reliability between the first measurements (data of the total sample) and the second measurements. No statistically significant differences were observed between both measurements (*p* > 0.05).

The inter-rater reliability of the obtained measurements was analyzed utilizing the intraclass correlation coefficient (ICC). ICC values greater than 0.90 indicated the excellent reliability of the repeated measurements.

### 3.3. Changes in Condylar Volume (mm^3^) before and after Twin Block Therapy with and without LLLT

Table 2 shows a comparison of condylar volume (mm^3^) within each group before (T1) and after (T2) TB therapy in both laser and control groups by applying a paired *t*-test. A statistically significant increase (*p* ≤ 0.05) in condylar volume in the laser group on both the right (213.3 mm^3^) and left (231 mm^3^) sides was observed. Similarly, the control group also demonstrated a significant increase (*p* ≤ 0.05) on the right (244.2 mm^3^) and left (225.2 mm^3^) sides after TB therapy. Table 3 shows a comparison of the change in condylar volume (T1-T2) measurements between laser and control groups using an independent sample *t*-test. The results showed insignificant differences between the groups on both the right and left sides (*p* > 0.05).

### 3.4. Changes in CBCT Measurements (mm) of Condylar Position before and after Twin Block Therapy with and without LLLT

Table 4 shows a comparison of the CBCT measurements of condylar position before and after TB therapy in the control group using a paired *t*-test. The results show no statistically significant change (*p* > 0.05) in both the right and left sides in the vertical condylar positions (0.4 mm and 0.4 mm, respectively) after TB therapy. Conversely, there was a statistically significant change (*p* ≤ 0.05) in both the right and left sides in the anteroposterior (0.7 mm and 0.8 mm, respectively) and mediolateral condylar positions (0.7 mm and 0.8 mm, respectively) after TB therapy.

Table 5 shows a comparison of CBCT measurements (mm) of condylar position before and after TB therapy in the laser group using a paired *t*-test. The results explain no statistically significant difference (*p* > 0.05) in both the right and left sides in the vertical condylar position (0.5 mm and 0.4 mm, respectively). On the other hand, there are statistically significant changes (*p* > 0.05) in the both right and left sides in the anteroposterior (0.7 mm and 0.9 mm, respectively) and mediolateral condylar positions (0.9 mm and 0.8 mm, respectively) after therapy.

Table 6 shows a comparison of the changes (T1-T2) in condylar position between laser and control groups using an independent sample *t*-test. On the right side, no statistically significant differences (*p* > 0.05) are evident in the anteroposterior (0.7 mm and 0.8 mm in laser and control groups, respectively), mediolateral (0.9 mm and 1.1 mm in laser and control groups, respectively) and vertical (0.5 mm and 0.4 mm in laser and control groups, respectively) condyle positions after TB therapy. Similarly, for the left side, there are no statistically significant differences (*p* > 0.05) in anteroposterior (0.9 mm and 1.1 mm in laser and control groups, respectively), mediolateral (0.8 mm and 0.9 mm in laser and control groups, respectively) and vertical condylar positions (0.4 mm and 0.4 mm in both laser and control groups, respectively) after TB therapy.

### 3.5. Changes in Cephalometric Skeletal Measurements before and after Twin Block Therapy with and without LLLT

Table 7 presents a comparison of the lateral cephalometric skeletal measurements before and after TB therapy in the control group using a paired *t*-test. The results showed no statistically significant differences (*p* > 0.05) in SNA (°) and CO-A (mm) measurements after therapy. However, a statistically significant increase (*p* ≤ 0.05) in the anteroposterior skeletal position measurements of the mandible was observed, as shown by ANB (°) and SNB (°) measurements. Similarly, mandibular effective length, Co–Gn (mm), ramus length (mm), and corpus length (mm) showed a statistically significant increase after TB therapy.

Table 8 demonstrates a comparison of cephalometric skeletal measurements before and after TB therapy in the laser group using a paired *t*-test. The results showed no statistically significant differences (*p* > 0.05) in measurements for SNA (°) and CO-A (mm) after therapy. On the contrary, a statistically significant increase (*p* ≤ 0.05) in the anteroposterior skeletal position of the mandible was observed, as shown by ANB (°) and SNB (°) angles. Correspondingly, mandibular effective, ramus, and corpus length showed a statistically significant increase after therapy. Table 9 shows a comparison of the changes in skeletal (T1-T2) measurements between laser and control groups using an independent sample *t*-test. There were nonsignificant differences between the groups regarding all tested skeletal measurements (*p* > 0.05).

## 4. Discussions

Class II malocclusions treated by the functional orthopedic approach are a matter of ongoing controversy due to the lack of consensus regarding the possibility of accentuating mandibular and/or condylar growth in a predictable manner. In the present investigation, the null hypothesis was not rejected since the changes subsequent to TB therapy, with and without LLLT, were not considerably different. Furthermore, all participating patients were treated using a TB appliance, according to the decided treatment plan, as the first stage for skeletal Class II patients presenting with mandibular retrognathism [3,6].

In the current study, an assessment of skeletal age was performed via evaluation of CVMS, as per the modified technique of Baccetti et al. [29]. The advantage of this version is that the mandibular skeletal maturity can be appraised on a single lateral cephalogram by analysis of only the second, third and fourth cervical vertebrae. The patients of both groups selected were at CS2-CS3 due to the initiation and peak of a growth spurt, so as to achieve maximum treatment effects using TB appliances. According to Baccetti et al. [31], maximum therapeutic effects are obtained if a mandibular growth spurt is included, as it can induce more favorable mandibular skeletal modifications. Furthermore, it was suggested that the optimal treatment time for TB appliance therapy is during or slightly following the onset of the pubertal peak [31].

Patients undergoing orthodontic treatment often complain about the length of the treatment time [25]. Recently, LLLT has been used to promote bone healing following fracture and mandibular distraction osteogenesis, for stimulating condylar cartilage and to reduce functional treatment time [24,26,27,28]. The results of those previous studies confirm that LLLT has a positive effect on the percentage of newly formed bone [24]. Furthermore, it is also found that low-level laser irradiations could accelerate orthodontic tooth movement within 2–3 months [21]. Previous studies evaluating the effect of LLLT to stimulate the condylar cartilage have done so on experimental animals [24,26,27,28]. Khadra et al. [34] demonstrated that LLLT may enhance bone formation in calvarial bone defects in rats. Seifi et al. [28], through their study, concluded that LLLT could promote condylar growth and cause mandibular advancement in experimental rats. Abtahi et al. [24] showed that LLLT during mandibular advancement in rabbits enhanced bone formation in the condylar region. The significant biostimulatory effect of LLLT mainly depends on the interaction of the tissues with light energy. The exact mechanism and laser energy density values for condylar biostimulatory effects and bone cell activities remain inconclusive. However, they may be multifactorial and include a series of processes such as the promotion of angiogenesis, osteogenic cell proliferation and differentiation, collagen production, mitochondrial respiration, and ATP synthesis [34].

Different types of lasers, including helium–neon, gallium–aluminum–arsenide, and gallium–arsenide, have been used at different doses, wavelengths and treatment schedules to obtain the desired effects [35,36]. In the present study, we used a gallium–aluminum–arsenide (GaAlAs) diode laser, which is known to have a high penetration depth compared to other laser types and thus presents as a non-penetrative tool of great efficiency. To the best of the author’s current understanding, no human data are available regarding this issue. Therefore, it was a matter of interest to incorporate LLLT during the functional treatment of patients with skeletal class II malocclusions. The present protocol of LLLT is clinically accepted and frequently utilized as a modality for the management of Temporomandibular disorders (TMD) [33].

In the orthodontic literature, cephalometric and panoramic radiographs are common techniques for TMJ evaluation and treatment outcomes because of their accessibility and availability, ease of use, low radiation doses, and low cost [14]. However, the efficacy of 2D radiographic imaging is doubtful due to the disparity created by patients’ head position, anatomic superimposition and magnification, in addition to the differences between the left and right sides [37]. CBCT is potentially useful to study TMJ development during the growing period [38] and is deemed necessary for 3D volumetric analysis. CBCT was used as a supplementary diagnostic aid where traditional radiographic approaches failed to provide outcomes for which imaging was performed [34]. CBCT was operated following the radiation dosage principle of “as low as diagnostically acceptable” (ALADA), which is a modification of “as low as reasonably achievable” (ALARA). Depending on the scan indication, the appropriate field of view (FOV), mAs, and kVp settings and high-resolution parameters are selected to acquire a diagnostically acceptable and interpretable image. Furthermore, the low-dosage CT protocols available nowadays have the same/similar amount of dosage in comparison to CBCT [35].

### 4.1. Condylar Volumetric 3D Analysis after TB Therapy with and without LLLT

The present 3D volumetric analysis showed significantly enhanced condylar volume in both laser and control groups following the use of a TB appliance. Currently, changes in condylar volume were determined by superimposition of pre- and post-TB treatment CBCT images on the foramen mentale and antegonial notch bilaterally. Once the images are superimposed, a 0.1mm imaginary line tangential to the distal slope of the coronoid process was established to determine the condylar area to be measured. The part of the mandible located above the imaginary line was determined for the volumetric analysis of the mandibular condyle. A similar technique was also used in the studies of Bayram et al. [17] and Yildirim et al. [16]. On the contrary, the present technique was different from that of the previously reported technique by Tecco et al. [39], in which the condylar head was used for volumetric assessment. The area to be measured was tracked where its section passed from an “elipsoidal” to a more “circular” shape.

However, the present findings are in accordance with the study by Yildirim et al. [16], who concluded that condylar CBCT volume and inter condylar distance increased after TB therapy without LLLT. Furthermore, the present results concur with those of Vedavathi et al. [15], who concluded that condylar CBCT volume increased by 310.4 mm^3^ after TB therapy without LLLT, slightly more on the left side compared to the right side, but not statistically different. In addition, Elfeky et al. [9] reported a statistically significant change in condylar length, width, and height on both the right and left sides after TB therapy without laser application. Nevertheless, following TB therapy, the current changes in 3D condylar volume in both laser and non-laser groups were comparable, with no major difference.

Alternatively, parallel findings were observed in several experimental studies concerning the changes in condylar morphology following LLLT. However, a comparison with these reports is difficult due to their experimental nature in animals. Seifi et al. [28] reported that 904 nm of LLLT irradiation stimulated condylar growth and facilitated mandibular advancement in experimental rats. Likewise, Abtahi et al. [24] reported that the application of 630 nm of LLLT during mandibular advancement in rabbits demonstrated enhanced bone formation in the condylar region. Furthermore, Saafan et al. [27] observed that 870 nm of LLLT with an energy density of 180 J/cm^2^ enhanced mandibular growth in rabbits’ TMJ area. In contrast, El-Bialy et al. [26] compared light-emitting diodes and LLLT on mandibular condylar growth with or without functional appliances in experimental rats and found the former to have an enhanced stimulatory effect on the mandibular condyles.

According to Nota et al. [40], a different pattern of increase in condylar volume was observed between males and females. The authors postulated that condylar volumetric increases seem to stop between the ages of 17 and 21 years in females, while they can continue at least until the end of the 17–21-year period in males. This significant difference between males and females was related to the disparity in the genome among individuals with different genders. From a clinical point of view, this outcome possibly suggests the stability of the treatment outcomes by interceptive orthodontics in male and female subjects [41].

### 4.2. Condylar Position Subsequent to TB Therapy with and without LLLT

In the present study, treatment via a TB appliance, with and without LLLT, showed a forward displacement of the condyles on both the right and left sides that could be related to the forward positioning of the mandibular arch by the TB appliance [7,8,9]. In both groups, a significant advancement was noticed in anteroposterior condylar position on both the right and left sides. Concerning the mediolateral condylar position, there was a statistically significant lateral movement in both patients treated with or without the laser that was evident on both the right and left sides. In contrast, the present findings revealed nonsignificant changes regarding the vertical condylar position, following TB therapy within each group, with or without LLLT.

The present findings are consistent with the cephalometric study of Baccetti et al. [31], CBCT studies of Elfeky et al. [9] and Liu et al. [12], and Magnetic Resonance Imaging (MRI) studies of Chavan et al. [8] and Arat et al. [7]. However, it is important to note that, although these positional condylar changes were statistically significant, they require careful clinical interpretation. In general, the majority of the functional appliances are constructed to promote forward mandibular growth by encouraging the functional displacement of the mandibular condyles downward and forward in the glenoid fossa. This is stabilized by the upward and backward pull of the muscles supporting the mandible [3,4,5,6] and thus adaptive remodeling can occur on both articular surfaces of the TMJ so as to improve the mandibular placement corresponding to maxilla [8].

In the current study, the changes observed in mandibular condylar volume and position in both laser and control groups following TB therapy were comparable, with no significant differences. This might designate that the effects of LLLT with TB therapy, using the current parameters and protocol, are debatable and necessitate supplementary clinical estimates.

Unfortunately, however, there are no clinical studies that address the effects of LLLT on condylar volume and position during functional orthopedic therapy, which makes a comparison with the present findings complicated. Once more, it is important to note that the abovementioned findings after TB therapy, with and without LLLT, are statistically noticeable, but their clinical consequence should be interpreted with caution.

### 4.3. Skeletal Changes

The secondary outcome of the current study was to compare the skeletal effects of TB, with and without LLLT, in patients with Class II malocclusions and mandibular retrognathism. Functional appliances were proposed to produce a distally directed force to maxilla as the mandible was repositioned forward. However, in the available literature, controversial outcomes are reported for the restraining effect of TB therapy on maxilla. In contrast, few studies show maxillary restriction following TB functional therapy [2,3,4,5,6,30].

In the current study, based on a comparison with baseline values, there were no significant changes in both the effective length and sagittal position of maxilla with and without the laser. These outcomes do not advocate any significant headgear effect associated with TB therapy. Analogous results were also emphasized by Clark [30], Elfeky et al. [9], and Yildirim et al. [16] without LLLT. However, the current findings are not compatible with those of Trenouth [42], and O’Brien et al. [2]. In those reports, it is not clear whether their sample included Class II patients with normal maxillary dimensions.

In the present research, on the other hand, an improvement in mandibular retrognathism was demonstrated following nine months of TB therapy. The effective mandibular length was increased, in both laser and control groups, which are desirable outcomes of treatment with functional therapy. Moreover, ramus and corpus lengths were augmented, with and without the laser. In the same way, without laser application, others also reported significantly enhanced mandibular growth with a TB appliance. In accordance with the current findings, Yildirim et al. [16] reported increased condylion–gnathion measurements after TB therapy. Correspondingly, Baysal and Uysal [43] reported that TB therapy resulted in a 3.37 mm additional increase in effective mandibular length. Moreover, after 12–16 months of TB therapy, an increase of between 1.46 and 4.75 mm in mandibular length was also reported by Baccetti et al. [31], Elfeky et al. [9] and O’Brien et al. [2]. Additionally, Jena and Duggal [32] had compatible results, with 1.98 mm of extra mandibular growth.

The current enhanced mandibular skeletal changes, in both groups, could be attributed to the observed significant changes in mandibular position, as evidenced by the favorable changes in SNB angle. Interestingly, these findings, over a shorter treatment period, are parallel to other studies that showed analogous outcomes following TB therapy without a laser, which is advantageous to patients with mandibular retrognathism [2,9,16,30,31,42,43,44].

Concerning the present inter-jaw relation, the treatment effects were mainly created by mandibular changes, as maxillary measurements were not affected by TB therapy. The current results clarified a significant improvement in the facial profile and a reduction in the severity of Class II skeletal pattern through the significant decrease in the ANB° angle in laser and control groups. These results are in harmony with other investigations [2,9,16,30,31,42,43,44]. However, a comparison of these effect changes yielded no significant difference between both groups with or without LLLT. Accordingly, it might be concluded that the observed sagittal changes could be achieved with TB therapy regardless of the inclusion of laser therapy.

However, it is noteworthy that the majority of the abovementioned studies observed a significant increase when compared with untreated control groups. Moreover, the present values were dependent on a comparison with pre-treatment values and natural growth effects must be considered.

The limitations of the current study could include the absence of an untreated control group to confirm the present findings and to exclude any possible effects of the natural growth pattern. However, the inclusion of an untreated control group was difficult for ethical reasons and, according to the available knowledge, there is no published CBCT information among Egyptians that could explain the natural growth changes in this regard. Moreover, evaluation was on a short-term basis, but needs further long-term investigations. Moreover, possible gender differences were not considered. It is recommended that additional randomized controlled trials with a larger sample size be carried out to further explore the effectiveness of LLLT during functional treatment. The current laser protocol was based on the recommendations for the treatment of TMD for only 3 months with TB therapy. Different results could be achieved if another longer protocol with different parameters is utilized in future clinical studies.

## 5. Conclusions

Considering the outcomes of the study, the following conclusions can be extracted:

1. Laser therapy, with the current parameters and protocol, accomplished no considerable effect on mandibular condylar volume and position following functional orthopaedic treatment of skeletal Class II malocclusions with a TB appliance.
2. The 3D condylar volume of both sides displayed a minor increase subsequent to the use of a TB appliance with and without LLLT.
3. Apart from LLLT, there are comparable changes in condylar positions, as evidenced by the minor forward and lateral movements, with no change in their vertical positions after TB therapy.
4. TB functional therapy resulted in a weighty improvement in skeletal profile and substantial skeletal changes in both mandibular dimensions and positions. However, these changes are hard to attribute to laser application.

## Figures and Tables

**Table 1:** Cephalometric reference landmarks and sagittal measurements used in the study.

Nasion (N)	The Most Anterior Midpoint of the Frontonasal Suture
Sella (S)	The central point of the pituitary fossa in the middle cranial fossa
Subspinale (point A)	The deepest midpoint on the anterior surface of maxilla.
Supramemtale (point B)	The deepest midpoint on the anterior surface of mandible.
Gonion (Go)	The right and left midpoint on the angle of the mandible, midway between ramus and corpus.
Gnathion (Gn)	The midway between menton and pogonion
Condylion (Co)	The most superior posterior point on the head of the condyle.
SNA°	The angle between 3 landmarks S, N, and A points, determining the anteroposterior position of the maxilla relative to the cranial base.
SNB°	The angle between 3 landmarks S, N and B points, determining the anteroposterior position of the mandible relative to the cranial base.
ANB°	The angle between three landmarks, A, N, and B points, determining the anteroposterior relation between maxilla and mandible relative to the anterior cranial base
Co–A (mm)	Effective midfacial length: the average of the bilateral linear distance between Co and A points
Co–Gn (mm)	Effective mandibular length: the linear distance between Co and Gn points.
Co–Go (mm)	Ramus length: the linear distance between Co and Go points.
Go–Gn (mm)	Corpus length: the linear distance between Go and Gn points.

**Table 2:** Comparison of condylar volume (mm^3^) before and after twin block therapy within each group using paired *t*-test.

Group		T1		T2		Mean Difference ± SE	95% CI		*p*-Value
		Mean	SD	Mean	SD		LL	UL	
Control group	Right side	1316.5	296.1	1560.7	336.8	244.2 ± 45.9	143.2	345.2	<0.001HS
	Left side	1267.1	252.3	1492.3	316.0	225.2 ± 45.4	125.2	325.2	<0.001HS
Laser group	Right side	1320.2	289.2	1533.4	296.6	213.3 ± 30.3	147.2	279.4	<0.001HS
	Left side	1202.8	331.4	1434.0	346.8	231.2 ± 39.5	145.2	317.2	<0.001HS

SD = standard deviation, CI = confidence interval, UL = upper limit, LL = lower limit SE = Standard Error, *n* = Number, *p* = probability level, T1 = before TB therapy, T2 = after TB therapy, HS = highly significant at *p* ≤ 0.001.

**Table 3:** Comparison of changes in condylar volume (T1-T2) measurements (mm^3^) between laser and control groups using independent sample *t*-test.

Condylar Volume	Control Group		Laser Group		*p*-Value
	Mean	SE	Mean	SE	
Right side (mm^3^)	244.2	45.9	213.3	30.3	1.000 NS
Left side (mm^3^)	225.2	45.4	231.2	39.5	0.650 NS

SE = Standard Error, *p* = probability level, *n* = number, T1 = before TB therapy, T2 = after TB therapy, NS = nonsignificant (*p* > 0.05).

**Table 4:** Comparison of CBCT measurements (mm) of condylar position before and after twin block therapy in control group using paired *t*-test.

Measurements		T1		T2		Mean Difference ± SE	95% CI		*p*-Value
		Mean	SD	Mean	SD		UL	LL	
Cdl-MSP (mm)	Right side	37.10	3.31	38.78	3.13	1.1 ± 0.3	1.9	0.8	0.035 S
	Left side	38.83	3.06	39.78	3.13	0.9 ± 0.4	2.9	0.2	0.026 S
Cds-FHP (mm)	Right side	2.17	2.74	2.59	2.70	0.4 ± 0.5	1.3	0.2	0.380 NS
	Left side	2.75	2.51	3.15	2.38	0.4 ± 0.4	1.4	0.1	0.375 NS
Cda-VP (mm)	Right side	6.68	3.68	7.40	3.87	0.8 ± 0.5	1.4	0.4	0.045 S
	Left side	7.48	4.07	8.66	3.68	1.1 ± 0.5	1.6	0.3	0.033 S

SD = standard deviation, CI = confidence interval, UL = upper limit, LL = lower limit, SE = Standard Error, Cds-FHP = vertical condylar position, Cda-VP = anteroposterior condylar position, Cdl-MSP = mediolateral condylar position, FHP = Frankfort Horizontal plane, VP = Vertical Plane, MSP = midsagittal plane, mm = millimeters, *p* = probability level, NS = nonsignificant, S = significant *p* ≤ 0.05, *n* = number, T1 = before TB therapy, T2 = after TB therapy.

**Table 5:** Comparison of CBCT measurements (mm) of condylar position before and after twin block therapy in laser group (*n* = 12) using paired *t*-test.

Measurements		T1		T2		Mean Difference ± SE	95% CI		*p*-Value
		Mean	SD	Mean	SD		UL	LL	
Cdl-MSP (mm)	Right side	37.90	1.65	38.57	1.73	0.9 ± 0.3	1.4	0.0	0.052 S
	Left side	37.51	1.72	38.29	2.13	0.8 ± 0.3	1.5	0.1	0.034 S
Cds-FHP (mm)	Right side	1.66	1.75	2.36	1.63	0.5 ± 0.2	1.1	0.3	0.102 NS
	Left side	1.88	1.84	2.28	1.65	0.4 ± 0.2	1.0	0.2	0.143 NS
Cda-VP (mm)	Right side	6.16	3.29	6.82	3.05	0.7 ± 0.2	1.2	0.2	0.013 S
	Left side	6.12	3.93	6.89	3.38	0.9 ± 0.4	1.3	0.1	0.042 S

SD = standard deviation, CI = confidence interval, UL = upper limit, LL = lower limit, SE = Standard Error, Cds-FHP = vertical condylar position, Cda-VP = anteroposterior condylar position, Cdl-MSP = mediolateral condylar position, FHP = Frankfort Horizontal plane, VP = Vertical Plane, MSP = midsagittal plane, mm = millimeters, *p* = probability level, NS = nonsignificant, S = significant *p* ≤ 0.05, *n* = number, T1 = before TB therapy, T2 = after TB therapy.

**Table 6:** Comparison of changes (T1-T2) in CBCT measurements (mm) of condylar position between laser and control groups using independent sample *t*-test.

Measurements		Control Group		Laser Group		*p*-Value
		Mean	SE	Mean	SE	
Cdl-MSP (mm)	Right side	1.1	0.3	0.9	0.3	0.406 NS
	Left side	0.9	0.4	0.8	0.3	0.225 NS
Cds-FHP (mm)	Right side	0.4	0.5	0.5	0.2	0.295 NS
	Left side	0.4	0.4	0.4	0.2	0.691 NS
Cda-VP (mm)	Right side	0.8	0.5	0.7	0.2	0.376 NS
	Left side	1.1	0.5	0.9	0.4	0.852 NS

SE = Standard Error, Cds-FHP = vertical condylar position, Cda-VP = anteroposterior condylar position, Cdl-MSP = mediolateral condylar position, FHP = Frankfort Horizontal plane, VP = Vertical Plane, MSP = midsagittal plane, *p* = probability level, NS = nonsignificant *p* > 0.05.

**Table 7:** Comparison of lateral cephalometric skeletal measurements before and after twin block therapy in control group using paired *t*-test.

Measurements	T1		T2		Mean Difference ± SE	95% CI		*p*-Value
	Mean	SD	Mean	SD		UL	LL	
SNA (°)	81.13	3.33	80.88	2.48	0.3 ± 0.4	0.5	0.1	0.503 NS
SNB (°)	73.29	3.05	76.83	2.54	3.5 ± 0.5	4.8	0.2	0.031 S
ANB (°)	7.83	1.61	4.54	1.03	3.7 ± 1.4	4.2	1.8	0.018 HS
Co–A (mm)	76.66	5.83	66.67	5.17	1.7 ± 0.7	1.9	0.5	0.113 NS
Co–Gn (mm)	94.38	6.51	97.71	6.30	2.8 ±0.4	3.1	1.3	0.001 HS
Co–Go (mm)	44.87	3.55	46.44	3.76	1.6 ± 0.4	2.4	0.8	0.001 HS
Go–Gn (mm)	62.73	4.88	64.76	4.52	1.3 ± 0.4	1.8	0.6	0.001 HS

SE = Standard Error, *p* = probability level, S = statistically significant at *p* ≤ 0.05, NS = nonsignificant (*p* > 0.05), HS = Highly Significant; SD = standard deviation, CI = confidence interval, UL = upper limit, LL = lower limit T1 = before TB therapy, T2 = after TB therapy, Sig = significance.

**Table 8:** Comparison of lateral cephalometric skeletal measurements before and after twin block therapy in laser group using paired *t*-test.

Measurements	T1		T2		Mean Difference ± SE	95% CI		*p*-Value
	Mean	SD	Mean	SD		UL	LL	
SNA (°)	81.47	2.89	80.68	2.68	0.4 ± 0.5	0.9	0.2	0.533 NS
SNB (°)	73.74	2.94	76.83	2.54	3.6 ± 0.4	4.7	0.1	0.041 S
ANB (°)	7.83	1.42	4.54	1.03	3.8 ± 1.3	4.3	1.7	0.028 S
Co–A (mm)	76.32	5.17	77.47	5.27	1.6 ± 0.8	1.9	0.5	0.123 NS
Co–Gn (mm)	94.18	5.79	97.71	6.30	2.9 ± 0.2	3.4	1.5	<0.001 HS
Co–Go (mm)	44.57	3.49	46.44	3.76	1.8 ± 0.2	2.7	0.8	<0.001 HS
Go–Gn (mm)	62.99	4.98	64.86	4.42	1.4 ± 0.3	1.9	0.5	<0.001 HS

*p* = probability level, S = statistically significant at *p* ≤ 0.05, NS = nonsignificant (*p* > 0.05), HS = Highly Significant; SE = Standard Error, SD = standard deviation, CI = confidence interval, UL = upper limit, LL = lower limit T1 = before TB therapy, T2 = after TB therapy.

**Table 9:** Comparison of changes (T1-T2) in skeletal measurements between laser and control groups using independent sample *t*-test.

Measurements	Control Group (*n* = 12)		Laser Group (*n* = 12)		*p*-Value
	Mean	SE	Mean	SE	
SNA (°)	0.3	0.4	0.4	0.5	0.347 NS
SNB (°)	3.5	0.5	3.6	0.4	0.728 NS
ANB (°)	3.7	1.4	3.8	1.3	0.728 NS
Co–A (mm)	1.7	0.7	1.6	0.8	0.247 NS
Co–Gn (mm)	2.8	0.4	2.9	0.2	0.689 NS
Co–Go (mm)	1.6	0.4	1.8	0.2	0.406 NS
Go–Gn (mm)	1.3	0.4	1.4	0.3	0.376 NS

SE = Standard Error, mm = millimeters, *p* = probability level, NS = nonsignificant *p* > 0.05, *n* = number.
